# Simulated Randomized Controlled Trial to Learn Critical Appraisal (SiRCA): A Randomized Controlled Study of Effectiveness Among Undergraduate Medical Students

**DOI:** 10.7759/cureus.19946

**Published:** 2021-11-27

**Authors:** Aneesh Basheer, Nayyar Iqbal, Stalin Prabakaran, Manjula Simiyon, Velavan Anandan

**Affiliations:** 1 General Medicine, DM Wayanad Institute of Medical Sciences, Wayanad, IND; 2 General Medicine, Pondicherry Institute of Medical Sciences, Pondicherry, IND; 3 Community Medicine, Pondicherry Institute of Medical Sciences, Pondicherry, IND; 4 Psychiatry, Pondicherry Institute of Medical Sciences, Pondicherry, IND

**Keywords:** medical education, critical appraisal, simulation, randomized controlled trial, evidence-based medicine

## Abstract

Introduction: The ideal method to teach evidence-based medicine (EBM) to medical students is unclear. We determined the effectiveness of a simulated randomized controlled trial (RCT) in improving critical appraisal and EBM skills among medical students compared to traditional training.

Methods: One hundred and eighteen medical students were randomized into two groups. Sixty-one students (immersion arm) were trained in critical appraisal using a simulated RCT aimed at determining efficacy of a “brainy pill” on ability to crack puzzles. Fifty-seven students (traditional group) were trained using a journal club with a checklist. Primary outcome of change in knowledge and skills of critical appraisal and EBM was determined by comparing scores on pre- and post-intervention Fresno tests.

Results: Mean age of students was 21.76 (SD - 0.78) years. Seventy (59.3%) were females and 48 (40.7%) males. Mean pre-test scores of traditional and immersion groups were 8.0 (SD - 4.88) and 9.31 (SD - 5.49) respectively and post-test scores were 50.2 (SD - 16.2) and 68.12 (SD - 14.72) respectively (post-intervention mean difference - 17.92; 95% CI 12.26 - 23.57; p<0.0000001). Odds of achieving 65% or more in post-intervention Fresno test score was significantly higher in immersion group (29.8% vs 8.2%; OR 4.76; 95% CI 1.62-13.97; p = 0.001). Perceived competence regarding EBM skills improved significantly in immersion group.

Conclusions: Simulated RCT is effective in imparting critical appraisal and EBM practice skills to medical students. Trainers should consider integrating and reinforcing this approach in EBM curriculum to make learning contextual and immersive.

## Introduction

Evidence-based medicine (EBM) has become the cornerstone of modern clinical practice. While the practice of EBM has been confined to clinicians, there is now a need to inculcate this behavior in medical students so that they become life-long practitioners of evidence-informed health care. EBM training is part of the undergraduate medical curriculum in many countries; however, its formal inclusion in the Indian medical curriculum and developing world has lagged behind others [1]. Now a great emphasis has been placed by the Indian regulatory body clearly spelling out that a graduate must be able to search and critically appraise medical literature effectively and apply it in clinical care [2].

Traditional EBM training has focused on delivery of much of the content in didactic form with a problem-based or case-based approach [3]. Most modules deal with developing answerable questions and searching literature. Developing skills of critical appraisal has been a part of the training in limited settings. Undergraduate EBM training is challenging because most students do not find the content relevant to their course. Examples used for training may be unrelated to the current phase of undergraduate learning and statistical elements may appear abstract. We therefore designed a new approach to training undergraduate students in EBM using a simulated randomized controlled trial and aimed to determine its effectiveness in imparting critical appraisal and other EBM competencies as compared to the traditional approach.

## Materials and methods

In 2016 a course on EBM was introduced at the Pondicherry Institute of Medical Sciences for the undergraduate medical students with the objective of sensitizing them to the need for evidence-based practice of health care [4]. The course was conducted over eight weeks during the third year of medical course and covered the key concepts of developing research questions, searching medical literature and critical appraisal of articles. Feedback on this course over the next couple of years revealed that while students understood the need for EBM and gained skills in literature search, complex issues such as critical appraisal and interpreting results of studies remained daunting tasks. In 2019, we modified the approach to teaching critical appraisal of randomized controlled trials using an immersion technique to discuss key issues. 

One hundred and fifty third-year medical students participated in the project after informed consent. A comprehensive course on EBM training was developed based on the ADDIE model [5] to cover eight weeks using five modules namely the principles and steps of EBM, framing foreground questions, effective literature search, critical appraisal of evidence and applying evidence to patients. While principles and steps of EBM were primarily didactic all other modules had hands-on elements with examples relevant to the phase of medical training of the students. For instance, scenarios used for developing search skills were from disciplines of ophthalmology and otorhinolaryngology which are the key specialties of training during the third year. Critical appraisal is usually taught in tandem with study designs and focuses on randomized controlled trials (RCT) as the prototype. We designed “Simulated RCT to learn Critical Appraisal (SiRCA)” as a tool to teach critical appraisal and interpretation of results of RCT. 

In this parallel arm, single-blind randomized controlled study, students were assigned randomly using computer-generated sequence of random numbers to two groups - the immersion group and the conventional group. Both groups completed a pre-test using the Fresno test at baseline [6]. The immersion group was trained in critical appraisal and basic statistical interpretation using the SiRCA, while the conventional group received didactic lectures with problem-based approaches and journal club discussion. At the end of the intervention, the students were assessed using the Fresno test to determine their level of EBM practice. Besides, feedback on the interventions was also taken. While the students could not be blinded to the intervention, the Fresno test assessor was blinded to the intervention allocation. 

A brief description of the SiRCA is as follows. The students who were assigned to the immersion group were informed about an opportunity to be volunteers for an RCT to test the efficacy of a hypothetical “brainy pill” versus placebo on the ability to crack puzzles. Following informed consent, they were randomized to two groups, during which computerized random sequence generation was demonstrated to students. Neither the students nor faculty were aware of the group to which a student was allocated thereby demonstrating concealed allocation. Students were then given one toffee each of similar color, shape and size from two different jars and asked to consume them, following which importance of blinding was discussed. An interactive journal club using conventional format of critical appraisal followed. Faculty collected height, weight and age of students to demonstrate how randomization would create groups with similar baseline characters. Later students were asked to find correct answers to three puzzles and return them in a sealed envelope. We hypothetically created sets of data from students’ responses (to demonstrate that brainy pill was effective) to calculate relative risk and p values. We also used the context to discuss how absence of few students would affect the results and demonstrated the concept of “intention to treat” analysis. 

Statistical analysis 

The primary outcome was mean difference between the groups in terms of post-intervention Fresno test scores. The proportion of students achieving 65% or more on post-intervention Fresno test and self-perceived improvement in confidence to critically appraise RCTs were secondary outcomes. Assuming that 5% of students in the traditional arm would achieve the cut-off of 65% on post-intervention Fresno test compared to 25% of students in the immersion arm, and power of 80% and alpha error of 5%, we estimated a sample size of 51 in each group (total 102). However, being an educational intervention, we included 118 willing students. We reported continuous data as means with standard deviations and categorical data as frequencies with percentages. Scores were compared between the two groups using student t test. Proportion of students achieving pre-specified cut-off was compared using the Chi square test. p values less than 0.05 were considered statistically significant.

## Results

**Figure 1:**
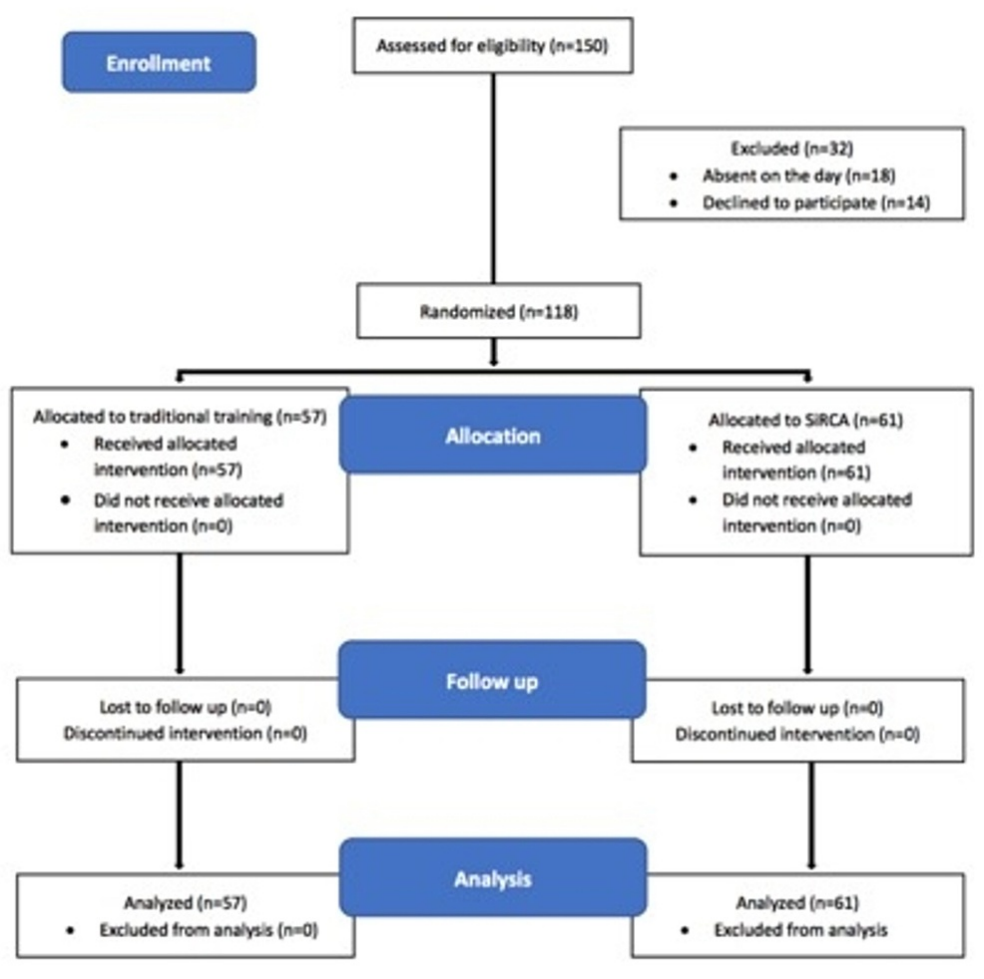
CONSORT flow diagram of participant recruitment, intervention and follow up CONSORT: CONsolidated Standards of Reporting Trials

Of the 150 students eligible to attend the EBM course, 118 took part in the study (Figure 1). The mean age of the students was 21.76 (SD - 0.78) years. There were 70 (59.3%) females and 48 (40.7%) males. All of them were in the third year of medical course and had attended the initial interactive EBM sessions dealing with concepts of EBM, framing foreground questions and literature search. 

Sixty-one students were randomly assigned to the immersion group while 57 were assigned to the conventional group. Major baseline characteristics of students in the two groups are summarized in Table 1.

**Table 1:** Baseline characteristics of students randomized to the immersion and conventional arms (n = 118)

Variable	Immersion group (n = 61)	Conventional group (n = 57)
Mean Age (SD)	21.76 (0.78)	20.89 (0.72)
Females (%)	37 (60.6%)	33 (57.8%)
Mean Percentage attendance at basic EBM sessions (SD)	94.5 (0.89)	95.3 (0.86)
Mean Pre-test score (SD)	9.31 (5.49)	8.0 (4.88)

The mean pre-test score of the conventional group was 8.0 (SD - 4.88) and that of the immersion group was 9.31 (SD - 5.49). After the interventions, their post-test scores were 50.2 (SD - 16.2) and 68.12 (SD - 14.72) respectively (mean difference - 17.92; 95% CI 12.26 - 23.57; t statistic - 6.2; p<0.0000001). While five out of 61 (8.2%) students in the conventional arm attained 65% or more in the Fresno test at the end of the intervention, 17 out of 57 (29.8%) of students in the immersion arm scored 65% or above with an odds ratio of 4.76 (95% CI 1.62-13.97; Chi square - 9.08; p = 0.001). The mean scores of students’ post-intervention in the questions pertaining to critical appraisal, interpretation of results and simple statistical calculations (of diagnostic test accuracy and effect estimates of RCTs) are summarized in Table 2. Feedback taken using retrospective pre- and post-test questionnaires on perceived competence regarding EBM skills before and after the interventions is summarized in Table 3. Mean scores for all domains showed a statistically significant improvement following the SiRCA.

**Table 2:** Post-intervention scores of students in the two groups on section of Fresno test pertaining to critical appraisal of RCTs and interpretation of results (including effect estimates and diagnostic test results) RCT - randomized controlled trial Immersion arm and Conventional arm values are mean scores with standard deviations in parentheses.

Competency	Immersion arm	Conventional arm	t statistic/degrees of freedom	p value
Critical appraisal of RCT	30 (8.4)	22.6 (9.8)	4.3/116	0.00002
Interpretation of results	23.4 (10.2)	20 (8.4)	1.9/116	0.049

**Table 3:** Comparison of perceived competence levels expressed as mean scores on Likert scale (1 - 4) before and after the immersion (SiRCA) based on retrospective pre-posttest questionnaire (n = 61) EBM - evidence based medicine RCT - randomized controlled trial Pre-intervention and Post-intervention values are mean Likert scale scores with standard deviations in parentheses

Competencies	Pre-intervention	Post-intervention	t statistic/degrees of freedom	p value
Interest in EBM	2.89 (0.63)	3.64 (0.71)	6.2/120	<0.0000001
Randomization	2.25 (0.70)	3.67 (0.45)	13.3/120	<0.0000001
Concealment of allocation	2.30 (0.67)	3.15 (0.57)	7.5/120	<0.0000001
Importance of baseline characteristics	2.00 (0.64)	3.70 (0.43)	17.2/120	<0.0000001
Blinding	2.36 (0.57)	3.80 (0.41)	16.0/120	<0.0000001
Follow up and attrition bias	2.17 (0.80)	3.55 (0.46)	11.7/120	<0.0000001
Intention to treat principle	1.88 (0.52)	3.69 (0.61)	17.6/120	<0.0000001
Steps of critical appraisal	1.76 (0.55)	3.47 (0.53)	17.5/120	<0.0000001
Confidence in critically appraising an RCT	2.26 (0.77)	3.66 (0.48)	12.1/120	<0.0000001

## Discussion

This randomized educational study tested the effectiveness of a novel immersive approach to inculcate critical appraisal skills among medical students during an EBM course. The SiRCA led to statistically significant improvements in knowledge and skills related to EBM practice including critical appraisal skills in particular compared to a conventional EBM module. Students also rated themselves more competent in several domains of EBM and confident in critical appraisal of RCT after the intervention. 

Traditionally EBM training has been centered around case-based or problem-based discussions and didactic lectures. However, the heart of EBM practice lies in the ability to critically appraise evidence found from an effective search. Several factors impede effective training of students in EBM, particularly critical appraisal skills. First, in most undergraduate curricula, especially in India, the emphasis on research is sparse. Second, undergraduate students find it difficult to understand the design and implementation of studies such as randomized controlled trials owing to the abstract nature of concepts like concealment of allocation, confounders, bias, intention to treat analysis and blinding [7]. Third, there is little by way of role modelling from mentors to demonstrate the place of critical appraisal of evidence in day-to-day clinical practice [8]. Fourth, the traditional method of training in critical appraisal using journal club stresses a checklist-based approach that leads only to recall level learning rather than deep learning. Finally, interpretation of results expressed as risk ratios, odds ratios and mean differences are viewed as formulae to be memorized instead of clinically meaningful expressions of effectiveness or harm. Students find them irrelevant to what they actually see and learn otherwise [9].

Although EBM has been in vogue for many decades now, there are very few studies that address the best method to train medical students in EBM. Early randomized controlled studies on postgraduate trainees revealed that isolated short EBM trainings may improve only knowledge in EBM while integrating EBM training with clinical training led to improved skills, knowledge and attitudes related to EBM [10]. Experts agree uniformly that training students in critical appraisal skills is the most difficult part of any EBM curriculum. Journal club discussions in large or small groups is a very commonly used method to develop critical appraisal skills [10,11]. However, the format of journal clubs is not standardized; the learning from such journal clubs may be influenced to a large extent by the mentors and their interests. Very often moderators divulge from a balanced appraisal to in-depth discussions of the subject content of their interest. A systematic review that examined the usefulness of journal clubs in EBM education concluded that there is insufficient evidence to suggest that it improves knowledge and skills related to EBM including critical appraisal and did not influence practice of EBM by health professionals [12]. Further, despite evidence that EBM training in any format leads to some changes in knowledge, skills and attitudes needed to practice EBM, no single method has been shown to be superior. The other lacuna in existing literature relates to the generalizability of findings; most randomized studies of interventions to train in EBM have been done on practicing clinicians, postgraduate trainees or interns. Experimental research on EBM training for undergraduate medical students is conspicuously limited [13], more so from Southeast Asian countries.

Hence we explored whether an innovative method of immersive experiential learning using a “simulated RCT” could improve understanding of critical appraisal and the overall knowledge and skills related to EBM practice among undergraduate medical students. Haidet and colleagues in 2002 described an interesting concept of “learning by doing” where they ventured to teach critical appraisal of RCTs by conducting a “mock RCT” in the class [14]. The authors concluded that the students felt this method enjoyable and stimulating to engage in critical appraisal of RCTs. However, the report did not mention data on actual improvements in outcomes; moreover, it focused on students’ perception of the new method. There was no comparison with traditional teaching. 

Subsequently Baker et al. demonstrated that mock RCT was a feasible and acceptable approach to teach critical appraisal and methodology of RCTs [15]. The authors described in detail the use of a simulated RCT in four different geographical sites with data collected using audience response technology (ART). The study included a heterogenous participant group ranging from experienced clinicians to students. The participants reported a better understanding of concepts of randomization, allocation concealment, baseline characteristics, blinding, follow up and bias. The data collected from the participants of the mock RCT through ART was used to discuss baseline data, bias and common statistical calculations. Authors used a before and after design to conclude that this approach improved learning related to EBM. Comparison of pre- and post-intervention scores of the cohort showed statistically significant improvement in knowledge of random sequence generation but not of the need for the same or the importance of determining attrition. But the confidence of all participants in identifying the basic steps of RCT and risk of bias increased significantly.

To the best of our knowledge, this is the first randomized controlled study to test the effectiveness of a simulated RCT to train participants on EBM in general and critical appraisal of RCT in particular. We used the approach by Haidet et al. [14] and Baker et al. [15] to design this immersive approach while improving the validity of the study by including a control group that received traditional teaching. Besides, this study included only undergraduate medical students wherein data on effective methods to teach EBM is limited. Therefore, our results have high external validity to the undergraduate medical students who are the future of evidence-based practice. While training in EBM was previously limited to clinicians and postgraduate trainees, many schools and universities have begun to realize that the EBM movement and spirit can only be sustained and accelerated by sensitizing and instilling its principles early in the training of medical students [16-18]. Five previous randomized controlled studies on undergraduate medical students comparing various innovative strategies (computer-based learning, problem-based learning, multi-disciplinary group learning, self-directed learning) with traditional methods found no difference in terms of improvement in knowledge, skills and attitudes towards EBM [19-23]. The results of our study that stand in contrast, therefore underscore a successful strategy to impart EBM skills to undergraduate medical students that many other methods failed to achieve. 

This study has limitations that require to be addressed in future endeavors. First, the improvement in EBM competencies was noted immediately after the intervention. As with any stand-alone or one-time educational intervention, the effect is unlikely to be retained in the long run. Second, although the outcomes were assessed using a standardized validated Fresno test of EBM competency, improvement in test scores may not mean better EBM skills in real practice. This outcome is consistent with a Kirkpatrick level 4 evaluation which is difficult to determine in many undergraduate educational programs [13,24]. Therefore, whether this intervention would inculcate the best EBM practices during their future years as doctors and whether this would in turn lead to better outcomes for their patients is unpredictable. Third, the prior experience and knowledge of students about statistics and study designs could have affected the results; while randomization would have balanced out such baseline variability between the two groups, we did not determine this specifically before the intervention. Fourth, the large differences observed between the pretest and post-test scores could have been influenced by the scant exposure to EBM and research at the undergraduate level in Indian medical schools. Therefore, the magnitude of improvements reported here may not be reproduced in the western schools where the baseline knowledge and skills of EBM may be higher. Finally, as with any educational intervention of this nature, results could be affected by contamination. We used geographically distinct areas for instruction of the two groups with outcome assessments immediate post-intervention. This minimized the potential for such contamination. Moreover, the marked difference between the scores of the two groups indicates that results are robust despite any negligible possibility of contamination. 

Nonetheless, the strong experimental design with blinding of assessors and the use of a standardized, validated and well-accepted instrument (Fresno test) to determine outcomes enhance the internal validity of the study. While we have not reported long-term outcomes in this paper, the simulated RCT has become a part of the EBM training at the institution with periodic reinforcements in a longitudinally integrated manner. This is likely to enable retention of skills and provide data to conclude about long-term effects.

## Conclusions

This randomised controlled study demonstrated significant improvements in competencies of undergraduate medical students in EBM and critical appraisal through the use of a simulated RCT (SiRCA). Students rated the experience interesting and felt that it improved their confidence in critical appraisal of randomized controlled studies as well as interpreting statistical data from studies. Longitudinal integration of SiRCA into EBM curriculum with periodic reinforcements is likely to have a long-term impact on EBM competencies of medical students with potential to translate into good EBM practices during real patient care settings.
